# Diverse Roles of NETosis in the Pathogenesis of Lupus

**DOI:** 10.3389/fimmu.2022.895216

**Published:** 2022-05-24

**Authors:** Meiying Wang, Tatsuya Ishikawa, Yupeng Lai, Dhiraj Nallapothula, Ram Raj Singh

**Affiliations:** ^1^ Autoimmunity and Tolerance Laboratory, Division of Rheumatology, Department of Medicine, David Geffen School of Medicine at University of California Los Angeles (UCLA), Los Angeles, CA, United States; ^2^ Department of Rheumatology and Immunology, Shenzhen Second People’s Hospital, The First Affiliated Hospital of Shenzhen University, Shenzhen, China; ^3^ Department of Pathology and Laboratory Medicine, David Geffen School of Medicine at UCLA, Los Angeles, CA, United States; ^4^ Molecular Toxicology Interdepartmental Program, David Geffen School of Medicine at UCLA, Los Angeles, CA, United States; ^5^ Jonsson Comprehensive Cancer Center, David Geffen School of Medicine at UCLA, Los Angeles, CA, United States

**Keywords:** NETosis, autoantigen, autoantibody, pathogenesis, systemic lupus erythematosus, self-tolerance, nephritis, clinical implications

## Abstract

NETosis is a form of neutrophil cell death during which extracellular fibrillary structures composed of cytosolic and granule proteins assembled on scaffolds of decondensed chromatin, called neutrophil extracellular traps (NETs), are released. NETs normally contribute to host immune defense. Accumulating evidence implicates aberrant NET production and/or reduced NET clearance, along with alterations of molecules involved in NETosis pathway, in humans and animals with lupus. The extruded nuclear antigens released by NET are a source of autoantigens, which can contribute to the breakdown of self-tolerance in lupus. Excessive NET can also promote the production of pro-inflammatory cytokine interferon-α, elicit direct cytotoxic effect on various renal cells, and cause capillary necrosis and podocyte loss. Additionally, NET can induce endothelial-to-mesenchymal transdifferentiation, which can promote activated myofibroblasts leading to extracellular matrix production. Thus, aberrant NETosis can play diverse roles, including autoantibody production, inflammation, and tissue damage, at different stages of lupus pathogenesis. Evidence suggests that treatments currently used in lupus may reduce NETosis, suggesting a potential utility of targeting NETosis to treat lupus. In fact, several approaches are being experimented to therapeutically target pathways of NETosis. Future studies should precisely delineate distinct roles of NETosis at different stages of lupus pathogenesis in humans, which would offer a rational basis for NETosis-targeting treatments in the clinic.

## Introduction

Compromised tolerance to self-antigens is an early step in the development of systemic lupus erythematosus (SLE) (1, 2). Among the diverse mechanisms that can mediate this process (1), NETosis is believed to play a major role. NETosis was first reported as a specialized form of cell death that occurs in neutrophils with neutrophil extracellular traps (NETs) released. This form of NETosis is now termed suicidal NETosis (3). Recently, it has been shown that NET formation can occur with preservation of neutrophilic functions, including phagocytosis and chemotaxis. This phenomenon is termed vital NETosis. Suicidal NETosis has been extensively evaluated and is considered as the classical NETosis, while research on vital NETosis is just unraveling (4). Stimuli and environment appear to be the most important determinants for neutrophils to undergo the suicidal NETosis or vital NETosis pathway (5). In this article, we evaluate the existing evidence on the role of NETosis in SLE (**Supplementary Figure S1**). We will mainly focus on suicidal NETosis, as little is known on the role of vital NETosis in SLE.

During the process of NETosis, NETs are released. NETs are large, extracellular, and fibrillary structures, which are composed of cytosolic and granule proteins that are assembled on a scaffold of decondensed chromatin (6). NETs, which can neutralize and kill bacteria, fungi, viruses and parasites, are the important first-line in host immune defense (7). However, if dysregulated, NETosis can contribute to the breakdown of self-tolerance and consequently lead to autoimmunity. In this article, we review pathways of NETosis and the role of NETs as a contributor to the loss of normal immune tolerance in lupus. We present evidence that supports excessive NET formation and reduced NET degradation in lupus and discuss multiple mechanisms whereby NET may contribute to lupus pathogenesis. Furthermore, we discuss clinical implications of NETosis and potential target of therapy by modulating NETosis in lupus.

## Formation and Function of NETs

**Figure 1:**
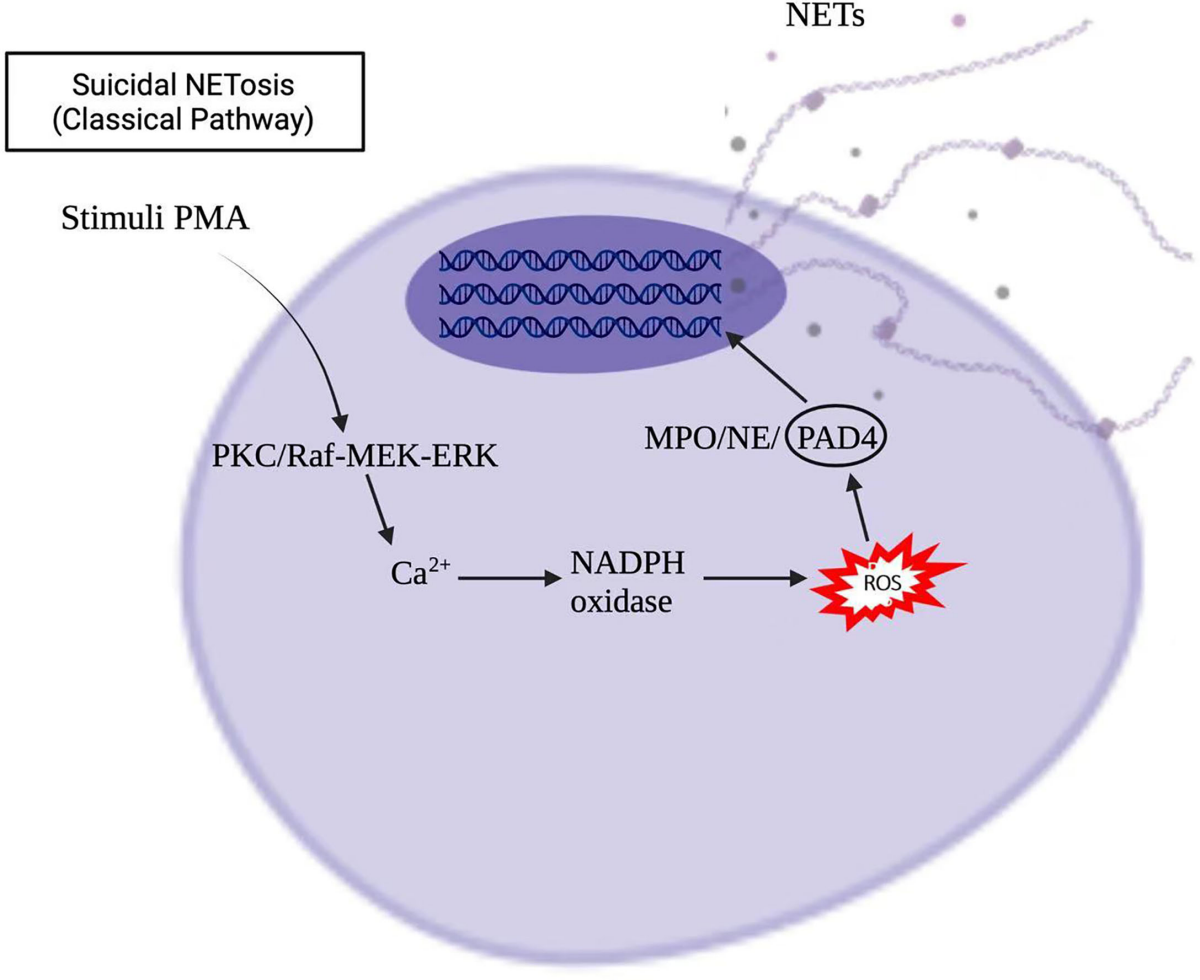
Phorbol 12-myristate 13-acetate (PMA) induced NETs formation in the suicidal NETosis.

**Figure 2:**
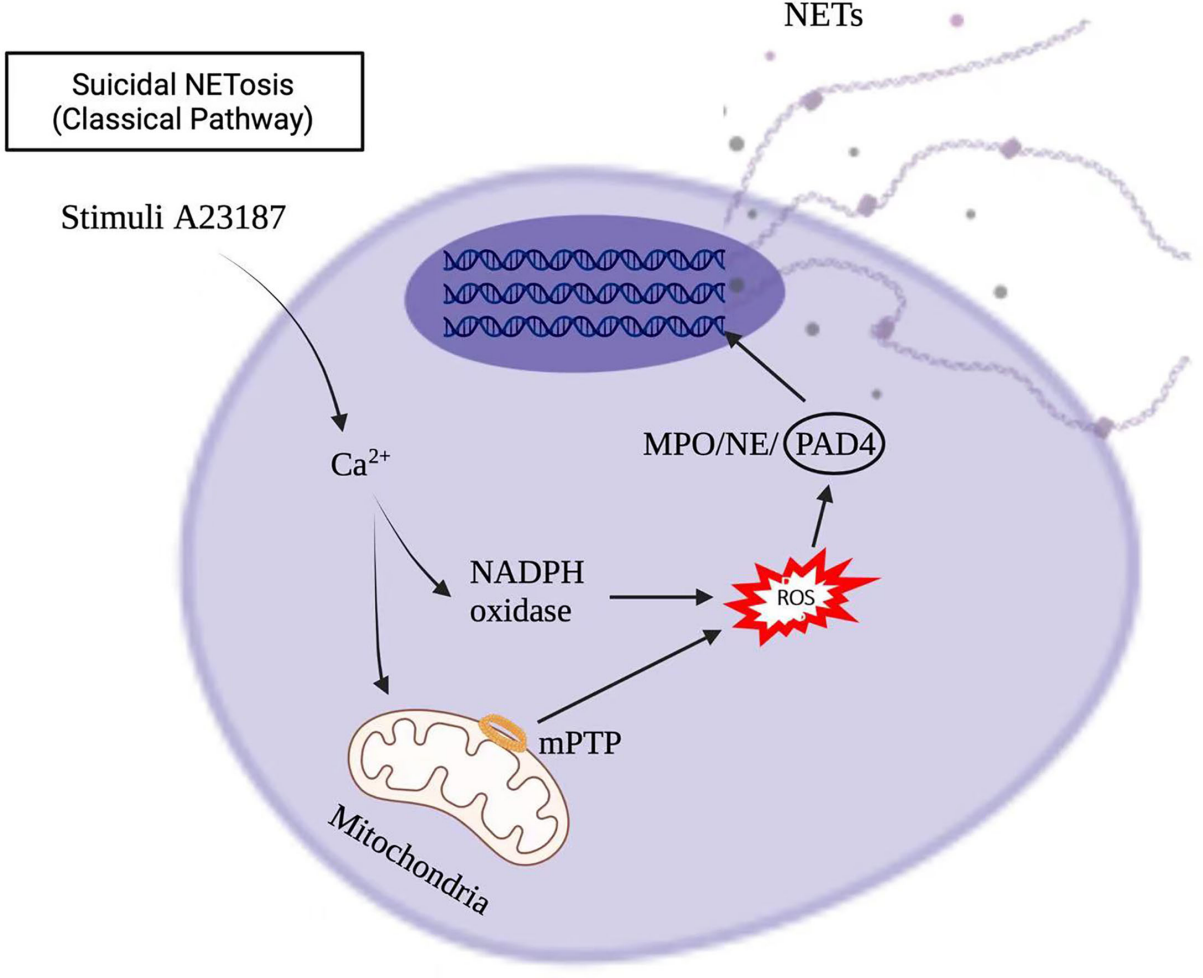
Calcium ionophore A23187 induced NETs formation in the suicidal NETosis.

**Figure 3:**
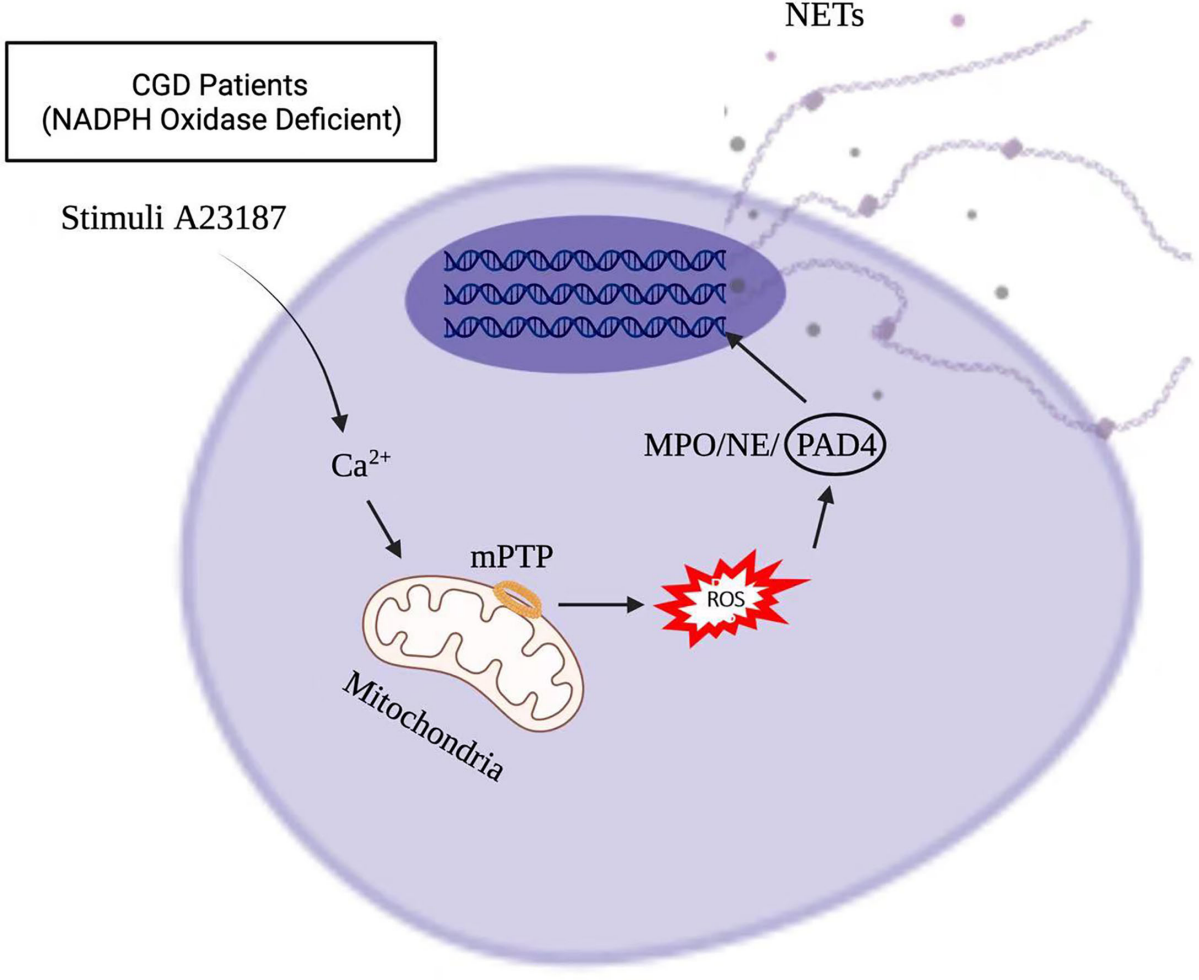
NET formation in X-linked chronic granulomatous disease (CGD) pathway, which is independent of NADPH oxidase.

Stimuli, such as infections, drugs, ultraviolet light, and hormones, trigger neutrophil activation through innate immune receptors, which activates downstream intracellular mediators that include protein kinase C/Raf-MEK-ERK, and therefore lead to calcium influx and reactive oxygen species (ROS) production (8, 9). ROS, produced by nicotinamide adenine dinucleotide phosphate hydrogen (NADPH) oxidase or mitochondria, activates myeloperoxidase (MPO), neutrophil elastase and protein-arginine deaminase type 4 (PAD4) to promote chromatin decondensation. Peptidylarginine deiminase 4 (PAD4)-dependent citrullination of histones induces decondensation of DNA resulting in a mixture of DNA and bactericidal proteins, including myeloperoxidase (MPO) and neutrophil elastase (NE), which are contained originally in intracytoplasmic granules. The increased cytosolic Ca^++^ can also act as a cofactor for PAD4, which is a nuclear enzyme that promotes the citrullination of histone, to facilitates the interaction with DNA (10), and lead to NETs formation. Vital NETosis is also a complex of DNA, MPO and NE. However, unlike suicidal NETosis, vital NETosis is ROS independent (5). Notably, different stimuli utilize different pathways to induce NET formation, as shown in **Figures 1**, **2** for pathways stimulated by phorbol 12-myristate 13-acetate (PMA) and calcium ionophore A23187, respectively (8). Patients with X-linked chronic granulomatous disease (CGD), who carry inactivating mutations of NADPH oxidase (NOX2), exhibit a specific pathway of NET formation, which is independent of NADPH oxidase (11) (**Figure 3**).

The function for NETs was first discovered in the context of infection and later in autoimmunology. During infection, DNA in NETs presents a rapid bactericidal activity by sequestering surface bound cations, disrupting membrane integrity and lysing bacterial cells (6, 12). *Via* post-apoptotic NETs, neutrophils around the marginal zone (MZ) of the spleen, acting as B cell helpers, generate an innate layer of antimicrobial immunoglobulin defense (13). In autoimmune disease, such as SLE, DNA covariates with large amounts of neutrophil proteins including LL37 (a cathelicidin antimicrobial peptide) and high-mobility group box 1 (HMGB1), to activate plasmacytoid DCs (pDCs). The SLE NETs activated pDCs produce high levels of interferon (IFN)-α in a DNA- and TLR9 (Toll-like receptor 9)–dependent manner. IFN-α, in turn, activates neutrophils, resulting in more NETs formation through a positive feedback cycle (14). SLE patients were found to develop autoantibodies to both the self-DNA and antimicrobial peptides in NETs, indicating that NETs could also serve as autoantigens to trigger B cell activation (15).

Studies have shown that LL37 and HMGB1, which are released during NETs formation, are autoantigens and are playing important roles in immunity and inflammation (16). They act as autoantigens, in combination with DNA. LL37-DNA complexes derived from NETs can directly trigger polyclonal B cell activation *via* TLR9, which can lead to increased antibody (Ab) production (17). HMGB1–DNA complexes may also be recognized by autoreactive B cells through B cell receptor–TLR7/9 interaction, resulting in autoAb production (18). Preclinical mouse models demonstrated that *in vitro* monocyte derived DCs take up DNA particles from neutrophils undergoing NETosis. Transfer of these DNA-loaded monocyte-derived DCs led to production of antibodies against dsDNA, MPO, and proteinase-3 (PR3) in mice. Autoantibody production was most significant when mice were injected with DNA-loaded monocyte-derived DCs that were exposed to NET-ting neutrophils. Thus, the extruded DNA from NET can also be more immunogenic than the whole apoptotic material (19, 20).

**Figure 4:**
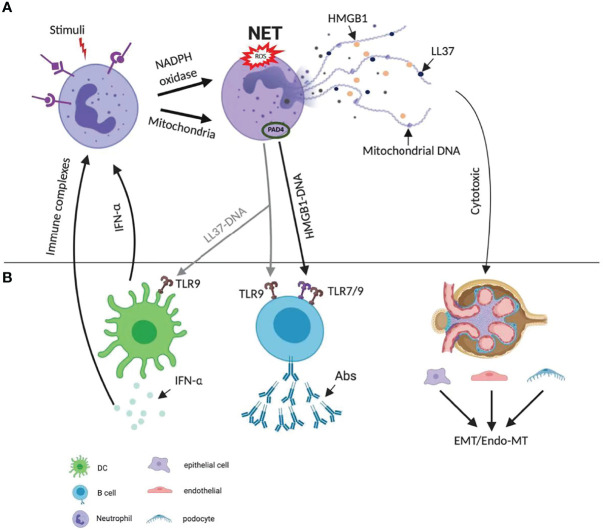
Diverse mechanisms of NETs’ role in pathogenesis of lupus. **(A)** NET formation starts with the activation of neutrophils through triggering of stimuli, which activate NADPH and mitochondria pathway. The latter promotes the citrullination of histone and facilitates their interaction with DNA. NETs are enriched in oxidized mitochondrial DNA, LL37 and HMGB1. **(B)** The extruded DNA from NETs is highly immunogenic. It activates plasmacytoid dendritic cells *via* TLR9 signaling, leading to the production of IFN-α. LL37-DNA complexes and DNA–HMGB1 complexes directly trigger polyclonal B cell activation *via* TLR9 or TLR7/9 and produce autoAbs. Both IFN-α and immune complex activate neutrophils, lead to more NETs formation and a positive feedback cycle. NETs can also have a direct cytotoxic effect on renal epithelial and endothelial cells *via* the externalization of histones, and therefore lead to epithelial-mesenchymal transition (EMT), endothelial-mesenchymal transition (Endo-MT). Abs, antibodies; EMT, epithelial-mesenchymal transition; Endo-MT, endothelial-mesenchymal transition; HMGB1, high-mobility group box 1; IFN, interferon; NADPH, nicotinamide adenine dinucleotide phosphate hydrogen; NET, neutrophil extracellular trap; PAD, peptidyl arginase deaminase; ROS, reactive oxygen species; TLR, toll-like receptor.

Besides the immunogenic effects, NET can also have a direct cytotoxic effect on human epithelial and endothelial cells *via* the externalization of histones. Incubation of epithelial and endothelial cells with histone type-IIA (which includes all types of histones) prevented cell growth, and provoked cytotoxicity in a concentration-dependent manner. These data confirm the cytotoxic capability of histones in NETs on epithelial and endothelial cells (21). Extracellular histones originating from NETs have been shown to promote capillary necrosis and podocyte loss, which can lead to proteinuria and crescent formation in severe glomerulonephritis (22). Since endothelial cells have a limited capacity to internalize NETs, the persistent extracellular NETs can induce vascular leakage through the degradation of intercellular junction protein VE-cadherin. Taken together, NETs have specialized immune-protective functions, but can also elicit autoimmunity and direct cytotoxic effects on renal cells. The interplay between neutrophils and other cell types is presented in **Figure 4**.

## Excessive NET Formation and Reduced NET Degradation in Lupus

### Animal Studies

Animal studies suggest several lines of evidence demonstrating altered NET formation and degradation in SLE. Neutrophils from lupus prone MRL/lpr mice present increased NETs formation compared with controls (23). Importantly, disrupting NETosis protected MRL/lpr and NZM mice against lupus by inhibiting PAD that is required for citrullination in NETs formation (23). PAD4 deficient (*Padi4–/–*) mice with FVB genetic background, displayed decreased autoantibodies, type I IFN responses, immune cell activation, vascular dysfunction, and NETs immunogenicity (24). In the Fc gamma receptor 2b deficient (*Fcgr2b*-/-) mouse model of lupus, renal ischemia reperfusion injury promoted NETs in peripheral blood neutrophils and kidneys, which was followed by glomerular immunoglobulin (Ig) deposition and increased serum anti-dsDNA Ab. NETs were detected in renal glomeruli using co-staining of MPO, neutrophil elastase, and citrullinated histone H3 for these mice (25) Together, these observations suggest an increased level of NETs in lupus pathogenesis.

Usually, NETs are timely removed to preclude the presentation of NET-associated self-Ag to immune cells. However, NETs degradation can be impaired in lupus *via* several mechanisms including dioxyribonuclease (Dnase) inhibitors, anti-NETs Ab, and defective phagocytosis (20). DNase has been shown to dissolve NETs *in vitro* (3). The knockout of *Dnase1* or of homologous *Dnase1l3*, both of which are responsible for DNase activity in serum, induces an SLE-like phenotype with autoAb and nephritis in otherwise normal mouse backgrounds (26, 27), which can be partly rescued by injecting an adenovirus encoding human *DNASE1L3* (27). Consistently, genetically lupus-prone MRL/lpr and NZB/W F1 mice carry hypomorphic variants of *Dnase1l3* (28). Macrophages have also been reported as important effector cells for NETs clearance; and lupus-prone mice with defective macrophages had increased anti-DNA Ab levels (29).

### Human Studies

Animal studies have been extended to human specimens, which suggests a possible role of NETs on SLE pathogenesis. Neutrophils from SLE patients exhibit an increased capability to form NETs compared to those from healthy people (30). NETs, which was visualized as web-like or granular structures co-staining with MPO, histone H2A, and DAPI, were observed in kidney biopsies from lupus patients (31). The locations of NETs components in inflamed kidneys, where are demonstrated as glomerular neutrophil infiltrates, were in the interstitium, around fibrinoid necrosis, and along the interlobular arterial wall in autoimmune small vessel vasculitis (32, 33).

Anti-dsDNA(+) SLE patients’ neutrophils were more prone to suffer from NETosis in comparison with anti-dsDNA(−) patients. Anti-dsDNA(+) patients displayed further altered levels of inflammation mediators, NETs, and cardiovascular risk. *In vitro*, Ig-dsDNA promoted NETosis on neutrophils, modulated the expression of inflammation and thrombosis-related molecules, and induced endothelial activation, suggesting a possible link between anti-dsDNA antibodies, the aberrant NETosis and increased cardiovascular risk in lupus (34). In resonance with animal studies suggesting a role of PAD4 and NETs formation in lupus (23, 24), the rs1635564 polymorphism of PAD4 is linked to nephritis in patients with SLE, further supporting the notion that PAD4 contributes to the pathogenesis of lupus nephritis (LN) (35). Notwithstanding, a recent study showed that adding an NADPH inhibitor (diphenyleneiodonium) or a PAD inhibitor (chloramidine) to SLE patients’ sera did not affect NET formation, which suggests that excessive NET formation in SLE may be independent of NADPH and PAD4 at least under certain circumstances. This study also suggested that the components of NETs were different in different disease conditions. SLE induced NETs had enrichment for oxidized mitochondrial DNA, whereas antineutrophil cytoplasmic antibody (ANCA)–associated vasculitis (AAV) induced NETs were enriched for citrullinated histones. These observations imply that therapeutic targets of NETs might be different for different diseases depending on the components of NETs (36).

Several lines of evidence indicate that the degradation of NETs can be impaired in patients with SLE *via* genetic changes, autoAb, complement, and macrophages. First, some patients with SLE carry the null mutations and hypomorphic variants of the DNase *DNASE1L3*. DNASE1L3 digests chromatin in microparticles released from apoptotic cells. Accordingly, people with null/hypomorphic variants of *DNASE1L3* have elevated levels of DNA in plasma, particularly in microparticles (27). The microparticles containing chromatin can drive ROS-independent NETs release, with glomerular deposition of NETs in actively SLE patients (37). Second, sera from some patients with active SLE, but not from those in remission, exhibit decreased ability to degrade NETs (38–40). The sera of these patients have high titers of anti-DNA, anti-histone, and other Ab that bind to NETs and thus protect NETs from degraded by DNase1 (38, 39). This agrees with the observation that anti-DNA Ab protect DNA from DNase fragmentation *in vitro* (3). Third, NETs activate the complement system, and the deposited C1q interferes with NETs degradation. IgG deposition on NETs also inhibited NETs degradation in tubules and glomeruli in the kidney of SLE patients (38, 39). Finally, NETs clearance by macrophages can be inhibited by ubiquitinated NETs proteins in patients with SLE (41).

## NET Can Promote Lupus *via* Multiple Mechanisms

### Animal Studies

Animal studies suggest that NETs can promote lupus *via* at least three different mechanisms, i.e., induction of specific autoantibodies, promotion of type I IFN secretion and induction of endothelial-to-mesenchymal transition (Endo-MT) (**Figure 4**).

First, myeloid DCs take up DNA and cytoplasmic Ag from NETotic neutrophils more efficiently than from neutrophils undergoing apoptosis or necrosis (42). The *in vivo* transfer of such NET-loaded myeloid DCs into naïve mice induced anti-dsDNA Ab and complement deposition and inflammation in kidneys. Consistent with a possible role of NETs in autoantibody production, treatment with polydatin that blocks ROS-mediated NET formation or with a JAK inhibitor tofacitinib that can target NETs formation *via* JAK/STAT pathway reduces serum autoantibodies levels and ameliorates lupus manifestations in the pristane-induced and MRL/lpr models (43, 44).

Second, evidence suggests a role of NETs in promoting lupus through increased type I IFN. For example, injection into mice of oxidized mitochondrial DNA released upon NET formation stimulates type I IFN signaling through a pathway dependent on the DNA sensor STING. This study showed that inhibition of mitochondrial ROS *in vivo* suppressed NETosis, type I IFN responses and lupus in MRL/lpr mice (45). Another study showed that lupus-prone MRL/lpr mice treated with mitochondria-targeted antioxidant (MitoQ) had reduced NET formation, decreased serum type I IFN, and reduced immune complex formation in kidneys, despite no change in serum autoantibody levels (46). The latter suggests that NETs may directly influence lupus pathogenesis *via* increasing type I IFN, and that this effect could be independent of NET’s effect on autoantibodies. Consistently, modulating NETs affected the expression of type I IFN-regulated genes in the MRL/lpr model of lupus nephritis (23).

Third, as discussed above, NETs can have direct cytotoxic effects on epithelial and endothelial cells via stimulation of pDCs. Evidence shows that excessive NETs can induce Endo-MT in cultured endothelial cells by NET-associated elastase. The correlation between the presence of NETs and Endo-MT in the nephritic MRL/lpr mice further suggests a role of NET-triggered Endo-MT in the pathogenesis of lupus (47). Notwithstanding, neutrophil elastase may not always be required in the execution of NETosis (48). Further studies are needed to decipher the role of neutrophil elastase in NET-induced Endo-MT.

#### Human Studies

In resonance with a possible role of NETs as a source of autoantigens, SLE NETs, compared to NETs from healthy individuals, contain increased amounts of acetylated and methylated histones; such post-translational modifications can create lupus autoantigens, such as acetylation of histone H4 at lysine 16 [reviewed in (30)]. Furthermore, NETs enriched in many SLE autoAg (49) have been shown to induce autoAb that lead to NETs-associated immune complexes (IC) formation (15), which promotes more NETosis, thus perpetuating a feedback cycle that results in excessive NETs formation in SLE patients (50). For example, ribonucleoprotein (RNP) IC that are prevalent in patients with SLE can induce NETosis (14). SLE neutrophils exposed to RNP IC induce the release of oxidized mitochondrial DNA, and NET enriched in oxidized mitochondrial DNA drive IFN-α production, thus can contribute to lupus disease (45, 51). NETs can also activate other immune cells. For example, lupus NETs can stimulate IL-1β and IL-18 secretion by LPS-primed macrophages from healthy individuals [reviewed in (30)]. Once secreted, IL-18 induces NET formation. NETs from healthy donors and SLE patients also increase calcium flux in macrophages from healthy donors and SLE patients (41). Finally, consistent with the idea that excessive NETs induce Endo-MT in murine lupus, the presence of NETs in the glomeruli of kidneys from patients with SLE is associated with the severity of proteinuria and glomerular Endo-MT (47). NETs in SLE have also been shown to promote vascular leakage, and induce endothelial cell apoptosis [reviewed in (30)]. Taken together, animal and human studies suggest that NETs may contribute to lupus pathogenesis via different mechanisms at different stages of disease, including early loss of self-tolerance, activation of other immune cells, inflammatory cytokine production, and endothelial damage.

## NETosis May Not Always Be Pathogenic in Lupus: Animal Studies

Although ample evidence suggests a pathogenic role of NETosis in lupus, a few animal studies suggest a no role or even a protective role of NETosis in lupus. The activation of the NADPH oxidase (Nox2) complex is required for NETs formation (52). Consequently, MRL/lpr mice that were rendered deficient in Nox2 had impaired NETs formation. Yet, Nox2-deficient MRL/lpr mice, which could not form NETs, had markedly exacerbated lupus disease. This suggests that NETosis may not contribute to SLE *in vivo*, and rather that Nox2 acts to inhibit disease pathogenesis in a way that is majorly NETs independent (53).

In addition, genetic deletion of PAD4, a distal mediator of NETs formation, also did not ameliorate loss of immune tolerance, immune activation or nephritis in MRL/lpr mice (54). In further support of this result, pharmacological inhibition of PAD did not improve disease in the Fas/lpr model of lupus, anti-glomerular basement membrane antibody-induced model of proliferative nephritis, and human-serum-transfer model of SLE (54). Compared to previous studies showing the effect of pan-PAD chemical inhibitors in ameliorating lupus in MRL/lpr mice (23) and of PAD4 and PAD2 deficiency in preventing lupus in FVB mice (24), PAD4 deficiency in MRL/lpr mice and pan-PAD chemical inhibitors in Fas/lpr did not improve lupus (54). The reasons for these discrepancies are not yet clear and may be related to the use of different mouse strains, the stage(s) of disease when the NET manipulations were performed, or the impact of partial inhibition versus complete inhibition of PAD activity. When it comes to the upstream and downstream mediators of NETs formation, both NOX2-deficient strain and PAD4-deficient strain exhibited impaired induction of NET formation, yet had elevated levels of antinuclear autoantibodies (ANAs) and exacerbated glomerulonephritis in the pristane-induced model of lupus. Corollary to this, treatment with Nox2-specific activators induced NETs formation, yet ameliorated pristane-induced lupus (55). Thus, while most studies thus far support the widely accepted notion that NETosis contributes to the pathogenesis of SLE, it is possible that NETs may have no role or even paradoxical roles in some model systems, in some patients, and in certain stages of disease.

## Clinical Implications of NET in Lupus

**Figure 5:**
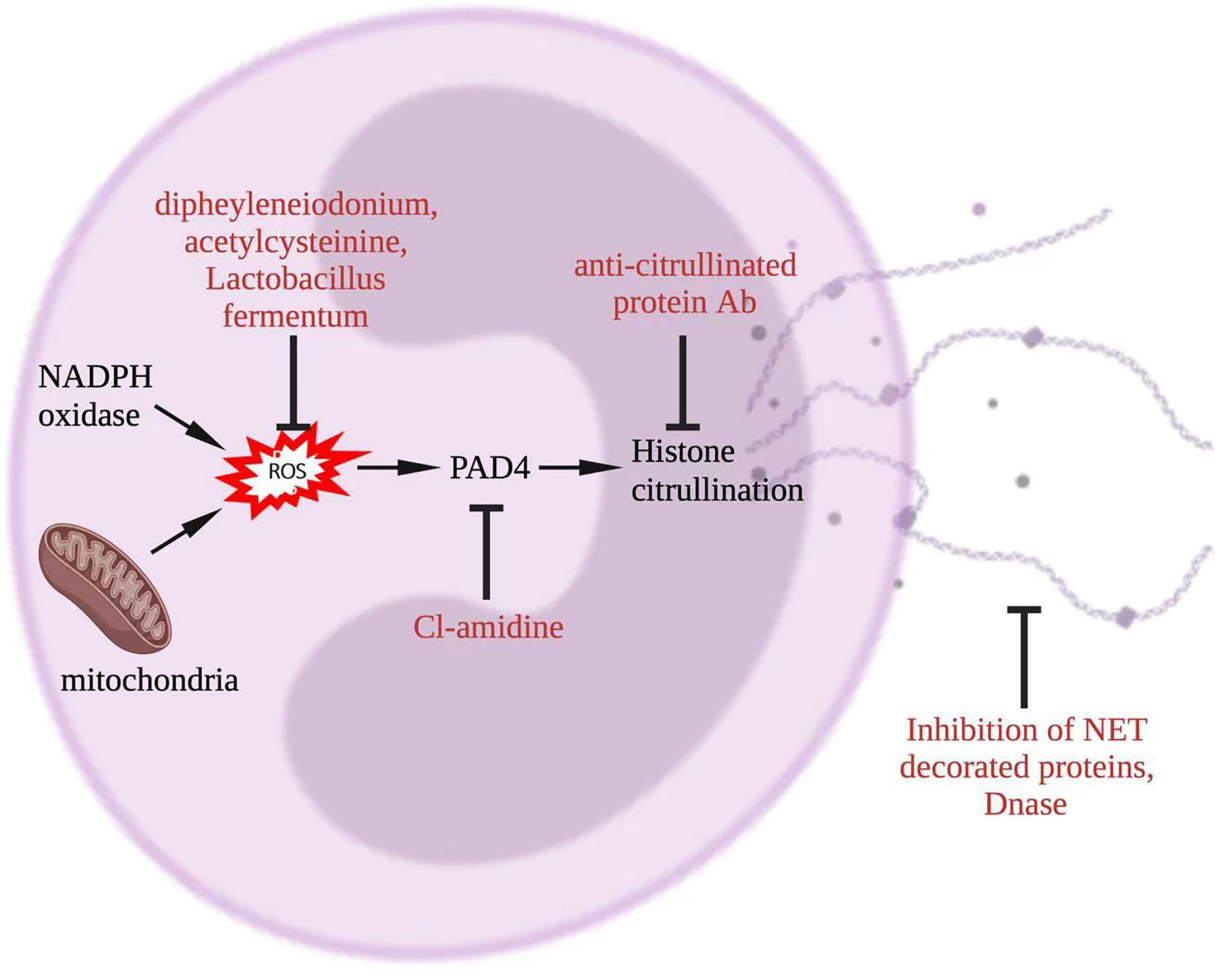
Potential approaches to target NET in lupus. To inhibit NETosis, ROS could be reduced by diphenyleneiodonium, acetylcysteine, or Lactobacillus fermentum, PAD4 could be targeted by CL-amidine, histone citrullination could be interfered through anti-citrullinated protein Abs, and NET degradation could be promoted by DNase. Additionally, NET-decorated proteins, such as interleukin-17A and tissue factor, could be targeted alternatively. Abs, antibodies; NADPH, nicotinamide adenine dinucleotide phosphate hydrogen; DNase, deoxyribonuclease; NET, neutrophil extracellular trap; PAD, peptidyl arginase deaminase; ROS, reactive oxygen species.

In this section, we will review potential links between current treatments for lupus and NETosis, and discuss possible ways to therapeutically target NETosis. The latter include targeting of molecules such as NADPH oxidase, MPO, and PAD4 which are involved in NETosis pathway to prevent or reduce NET formation, accelerating the degradation of NET, deactivating molecules that are downstream of NET, and secondarily inhibiting NETosis (**Figure 5**).

Hydroxychloroquine and corticosteroids, which are cornerstone of drug therapy in SLE, have been shown to decrease NET formation *in vitro* (20). Since autoAb and immune complexes can trigger NET formation, treatments that reduce autoAb could reduce NET formation (20). For example, treatment with a combination of rituximab and belimumab reduced autoAb levels and NET formation in patients with SLE (50).

Several approaches are being proposed to directly reduce the formation of NET and/or enhance their degradation (20). These approaches include targeting ROS with diphenyleneiodonium (56), targeting mitochondrial ROS with N-acetylcysteinine (57), inhibiting PAD enzymes by Cl-amidine (23), enhancing breakdown of NET with DNase1 (58), using DNASE1L3 to increase DNA digestion in apoptotic microparticles (27), and targeting histones with anti-citrullinated protein Ab (59). Some of these approaches are being tested in lupus in preclinical animal model and human studies. For example, lupus-prone MRL/lpr and NZM mice treated with PAD inhibitors, namely Cl-amidine and BB-Cl-amidine, had reduced NET formation and were protected from lupus (23). NADPH oxidase inhibitors can also suppress NET production. For example, bacterium Lactobacillus fermentum CECT5716 (LC40) protected kidneys in a mouse model of lupus by inhibiting NADPH oxidase activity (60). In a recent study, a Syk inhibitor fostamatinib attenuated NETs in neutrophils and reduced lupus characteristics (serum creatinine, proteinuria, and anti-dsDNA) in *Fcgr2b*-/- lupus mice (25).

Studies have shown that SLE NETs decorated with downstream molecules, including tissue factor and interleukin-17A (IL-17A), promoted thrombin generation and the fibrotic potential of cultured skin fibroblasts (61). In mouse model of lupus, mice lacking IL-17 were protected from the development of glomerulonephritis and had improved survival (62). This suggests that inhibition of IL-17A or tissue factor expressed on NET could be another potential therapeutic target.

In human SLE neutrophils, NETosis could be inhibited by adding a therapeutic anti-citrullinated protein Ab; this Ab also prevented NET-mediated organ damage in animal models of inflammation (59). JAK inhibitor tofacitinib, a drug approved to treat inflammatory diseases, modulated NET formation and ameliorated lupus in MRL/lpr mice (44), and is under clinical investigation in SLE patients (NCT02535689). Using a peptide inhibitor of complement C1, PA-dPEG24, to reduce NET formation by human neutrophils (63) offers another potential NETosis-based therapeutic approach.

In a recent study, 6-gingerol, the most abundant bioactive compound of ginger root, attenuated NET release in response to lupus- and APS-relevant stimuli, such as RNP ICs and aPL (APS IgG), in human neutrophils *in vitro*. Administration of 6-gingerol to mice reduces NET release in various models of lupus and APS, while also improving disease-relevant endpoints, such as autoantibody (anti-dsDNA and anti-β2GPI) formation and large-vein thrombosis (64).

A recent report identified inositol-requiring enzyme 1 α (IRE1α) as a critical mediator of lupus-derived immune complex–mediated NETosis *in vitro* (65). Importantly, pharmacological inhibition of IRE1α using KIRA6 reduced mitochondrial ROS formation, NET release (in both human neutrophils and a mouse model of lupus), and autoantibody formation in multiple lupus mouse models. Thus, inhibition of the IRE1α pathway could be an effective strategy for neutralizing NETosis in lupus.

Quantifying NET formation through the course of lupus disease may serve as a biomarker for disease activity, as suggested by studies showing increased circulating NET in patients with active SLE and reduced NET in SLE patients in remission (39, 50). However, currently there is no gold standard to measure NET formation (4, 66). The current strategies to detect NETs including microscopy, ELISA, immunoblotting, flow cytometry, and image-stream-based methods suffer from drawbacks such as being subjective, error-prone, non-specific and not being high throughput. This can be alleviated by new imaging based high throughput methods. For example, a novel imaging flow cytometry approach for the measurement of NETs both *in vitro* and in whole blood samples can provide an unbiased, accurate and rapid quantification of NET formation (67). Another study employed membrane-permeable and impermeable DNA dyes *in situ* to stain NET-forming cells, and used automated algorithm-driven single cell analysis of change in nuclear morphology, nuclear area and intensities to precisely detect NET-forming cells (68). Such high throughput approaches may provide a good platform to evaluate NETs as a surrogate marker of disease activity in the clinic and for the discovery of potential inhibitors of NET formation.

## Synopsis

Taken together, observations in humans and animals with SLE support the idea that excessive NETosis and reduced NET degradation play a role in autoAb production, inflammation, and tissue damage in lupus (**Table 1**). Impaired NETosis is believed to mark an early step in lupus pathogenesis. The extruded nuclear Ag released by NET serve as autoAg, and the failure to dismantle NET plays a role in the breakdown of normal immune tolerance. The persistent exposure of nuclear particles then activates immune cells, which facilitate immune response against self-Ag. Excessive NET can also induce Endo-MT in cultured endothelial cells, which contributes to activated myofibroblasts and extracellular matrix production. Thus, NETosis may play different pathogenic roles at different stages of lupus. However, a few animal studies suggest that NETosis may have no role at all or even a protective role in the development of lupus in certain model systems. These potentially dual, pathogenic versus protective roles, of NETs will need to be precisely delineated at different stages of lupus disease before NETosis-targeting treatments can be used in the clinic. Nonetheless, preclinical discoveries on mechanisms of NET formation, together with clinical findings in lupus, will pave the way for further investigations into targeting NET formation therapeutically and as a biomarker.

**Table 1:** Overview on the role of NETosis in the pathogenesis of lupus.

	Evidence/Comments
Animal studies	Increased NETosis in neutrophils from lupus mice (23); Reduced NET degradation (20); Hypomorphic variants of DNase gene in genetically lupus-prone mice (28).
Human data	Increased NETosis in SLE patient neutrophils (30); NETs visualized in lupus kidney biopsies (31); Microparticles and RNP-IC induce NETosis (14); Reduced ability to degrade NETs (38); SLE linked to DNase gene variants (27).
Implications for pathogenesis	Breakdown of self-tolerance, leading to autoAb production (42); NETs promote Type 1 IFN production (44); NETs can be directly cytotoxic to renal cells (21); NETs can induce Endo-MT (47).
Clinical implications	Therapeutic targets: agents to reduce the formation of NETs and/or enhance their degradation (20); agents that can target molecules that decorate NETs (61); inhibiting NETosis using antibodies (59) Potential biomarker of lupus activity (67).
Knowledge gaps	Human translation; Data on distinct disease-specific forms of NETs; Standard measurements for NETs using high throughput methods for clinic use.

DNase, deoxyribonuclease; Endo-MT, endothelial-mesenchymal transdifferentiation; IC, immune complexes; IFN, interferon; NET, neutrophil extracellular traps; RNP, ribonucleoprotein; SLE, systemic lupus erythematosus.

## Data Availability Statement

The raw data supporting the conclusions of this article will be made available by the authors, without undue reservation.

## Author Contributions

MW and RS developed the outline and objective of the article, MW wrote the first draft of the article. TI and DN assisted with literature review, preparation of figures, and writing. YL added part of the content. MW and RS revised the article critically for its intellectual content. All authors approved the final version to be published, and take responsibility for the integrity of the content covered in this article.

## Funding

This work was supported in part by the Science and Technology Program for Basic Research in Shenzhen (No. JCYJ20190809095811254 and JCYJ20200109140412476), Hospital Clinical Research Project (20213357002), NIH R01 AR050797, R01 AI080778, and R01 AR056465 grants.

## Conflict of Interest

The authors declare that the research was conducted in the absence of any commercial or financial relationships that could be construed as a potential conflict of interest.

## Publisher’s Note

All claims expressed in this article are solely those of the authors and do not necessarily represent those of their affiliated organizations, or those of the publisher, the editors and the reviewers. Any product that may be evaluated in this article, or claim that may be made by its manufacturer, is not guaranteed or endorsed by the publisher.
