# Supplementary material for: Diverse Roles of NETosis in the Pathogenesis of Lupus
Source: Front Immunol. 2022 May 24;13:895216. doi: 10.3389/fimmu.2022.895216 (PMC9170953; doi:10.3389/fimmu.2022.895216)
Supplement: Supplementary file 1 [file DataSheet_1.docx]

**Identification of studies via databases and registers**

Records removed *before screening*:

Duplicate records removed (n =66)

Records marked as ineligible by automation tools (n =0 )

Records removed for other reasons (n =0 )

Records identified from*:

Databases (n =248 )

Registers (n =0 )

**Identification**

Records screened

(n =182 )

Records excluded**

(n =53 )

Reports sought for retrieval

(n =129 )

Reports not retrieved

(n =28 )

**Screening**

Reports assessed for eligibility

(n =101 )

Reports excluded:

Reason 1. Repeated reviews (n =26 )

Reason 2. No Impact Factor (n =7 )

Studies included in review

(n =68 )

Reports of included studies

(n =68 )

**Included**

*Consider, if feasible to do so, reporting the number of records identified from each database or register searched (rather than the total number across all databases/registers).

**If automation tools were used, indicate how many records were excluded by a human and how many were excluded by automation tools.

*From:*  Page MJ, McKenzie JE, Bossuyt PM, Boutron I, Hoffmann TC, Mulrow CD, et al. The PRISMA 2020 statement: an updated guideline for reporting systematic reviews. BMJ 2021;372:n71. doi: 10.1136/bmj.n71

For more information, visit: <http://www.prisma-statement.org/>
